# Clinical Presentation of Coronavirus Disease 2019 (COVID-19) in Pregnant and Recently Pregnant People

**DOI:** 10.1097/AOG.0000000000004178

**Published:** 2020-10-06

**Authors:** Yalda Afshar, Stephanie L. Gaw, Valerie J. Flaherman, Brittany D. Chambers, Deborah Krakow, Vincenzo Berghella, Alireza A. Shamshirsaz, Adeline A. Boatin, Grace Aldrovandi, Andrea Greiner, Laura Riley, W. John Boscardin, Denise J. Jamieson, Vanessa L. Jacoby

**Affiliations:** Departments of Obstetrics and Gynecology and Pediatrics, University of California, Los Angeles, Los Angeles, California; the Departments of Obstetrics, Gynecology, and Reproductive Sciences, Pediatrics, Epidemiology and Biostatistics, and Medicine, University of California, San Francisco, San Francisco, California; the Department of Obstetrics and Gynecology, Thomas Jefferson University, Philadelphia, Pennsylvania; the Department of Obstetrics and Gynecology, Baylor College of Medicine, Houston, Texas; the Department of Obstetrics and Gynecology, Massachusetts General Hospital, Boston, Massachusetts; the Department of Obstetrics and Gynecology, University of Iowa, Iowa City, Iowa; the Department of Obstetrics and Gynecology, Weill Cornell Medical College, New York, New York; and the Department of Gynecology and Obstetrics, Emory University School of Medicine, Atlanta, Georgia.

## Abstract

**OBJECTIVE:**

To describe the clinical presentation, symptomology, and disease course of coronavirus disease 2019 (COVID-19) in pregnancy.

**METHODS:**

The PRIORITY (Pregnancy CoRonavIrus Outcomes RegIsTrY) study is an ongoing nationwide prospective cohort study of people in the United States who are pregnant or up to 6 weeks postpregnancy with known or suspected severe acute respiratory syndrome coronavirus 2 (SARS-CoV-2) infection. We analyzed the clinical presentation and disease course of COVID-19 in participants who tested positive for SARS-CoV-2 infection and reported symptoms at the time of testing.

**RESULTS:**

Of 991 participants enrolled from March 22, 2020, until July 10, 2020, 736 had symptoms of COVID-19 at the time of testing; 594 tested positive for SARS-CoV-2 infection and 142 tested negative in this symptomatic group. Mean age was 31.3 years (SD 5.1), and 37% will nulliparous. Ninety-five percent were outpatients. Participants who tested positive for SARS-CoV-2-infection were a geographically diverse cohort: 34% from the Northeast, 25% from the West, 21% from the South, and 18% from the Midwest. Thirty-one percent of study participants were Latina, and 9% were Black. The average gestational age at enrollment was 24.1 weeks, and 13% of participants were enrolled after pregnancy. The most prevalent first symptoms in the cohort of patients who tested positive for SARS-CoV-2 infection were cough (20%), sore throat (16%), body aches (12%), and fever (12%). Median time to symptom resolution was 37 days (95% CI 35–39). One quarter (25%) of participants who tested positive for SARS-CoV-2 infection had persistent symptoms 8 or more weeks after symptom onset.

**CONCLUSION:**

COVID-19 has a prolonged and nonspecific disease course during pregnancy and in the 6 weeks after pregnancy.

**CLINICAL TRIAL REGISTRATION:**

ClinicalTrials.gov, NCT04323839.

## Abstract 2

Coronavirus disease 2019 (COVID-19) has a prolonged course in ambulatory pregnant patients.

The emergence of coronavirus disease 2019 (COVID-19), caused by the novel severe acute respiratory syndrome coronavirus 2 (SARS-CoV-2), was described in Wuhan, China, in December 2019.^1^ By March 11, 2020, the World Health Organization had declared the outbreak of COVID-19 a pandemic.^2^

Despite the potential increased risks of COVID-19 for pregnant patients and their newborns, there remain significant gaps in knowledge on the clinical course of disease and the overall prognosis in this population. To date, studies of SARS-CoV-2 infection in pregnancy have primarily focused on hospitalized patients. Currently, there are limited data on longitudinal changes in symptoms over time and total duration of disease among the pregnant outpatient population, which includes the majority of patients with COVID-19. Data on clinical evolution are critical to guide and inform counseling and risk stratification for pregnant patients with COVID-19.

To evaluate the clinical course and disease outcomes of a nationally representative sample of pregnant patients with SARS-CoV-2 infection, we launched PRIORITY (Pregnancy CoRonavIrus Outcomes RegIsTrY) on March 22, 2020. PRIORITY is an ongoing prospective registry of pregnant and recently pregnant patients across the United States who are persons under investigation or have a confirmed SARS-CoV-2 test result. Participants are queried serially after enrollment about their test results and clinical symptoms. The objective of this analysis was to describe the clinical presentation of symptomatic pregnant people with SARS-CoV-2 infection over time.

## METHODS

**Fig. 1.**
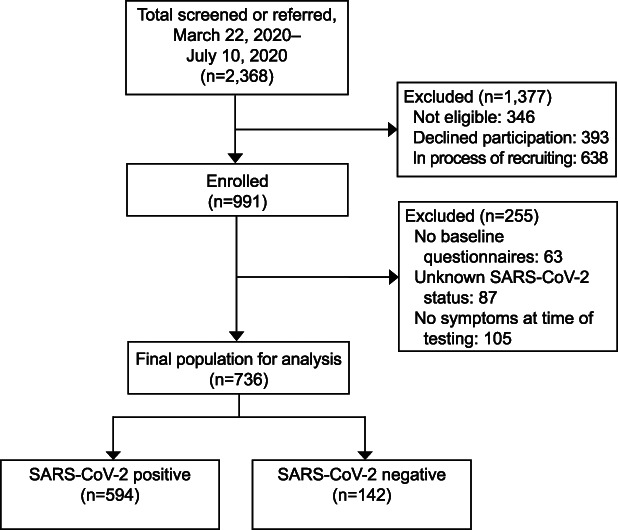
PRIORITY (Pregnancy CoRonavIrus Outcomes RegIsTrY) study population: screening, enrollment, and study population. SARS-CoV-2, severe acute respiratory syndrome coronavirus 2. Afshar. Clinic Presentation of COVID-19 in Pregnancy. Obstet Gynecol 2020.

PRIORITY is an ongoing prospective cohort study of pregnant or recently pregnant patients with known or suspected SARS-CoV-2 infection. The institutional review board approved the study (UCSF IRB# 20-30410, UCLA IRB# 20-000579). Informed consent was obtained from participants in accordance with institutional review board protocols as described below. Participants included in this study were enrolled in PRIORITY from March 22, 2020, until July 10, 2020 (Fig. 1).

Eligible participants included those aged 13 years or older who were pregnant or had been pregnant within the previous 6 weeks, had been diagnosed with or were being evaluated for (“person under investigation”) SARS-CoV-2 infection at any time since January 1, 2020, and were able and willing to give informed consent. There were no language restrictions, because consent was obtained through use of an official medical interpreter. Patients with COVID-19 who were in the intensive care unit on respiratory ventilators were eligible to enroll through proxy consent by the designated medical decision maker. If enrolled by a medical proxy, consent was obtained from the participant once she gained capacity. Written consent for Health Insurance Portability and Accountability Act (HIPAA) regulations and release of medical records was obtained electronically (or mail or fax) using DocuSign. We recruited through promotion with professional societies and networks of obstetric care professionals and with social media campaigns (see the PRIORITY fliers [in English and Spanish] at https://priority.ucsf.edu/healthcare-providers and the Reproductive Health Equity and Birth Justice CORE at https://birthjustice.ucsf.edu/reproductive-health-equity-and-birth-justice-core%C2%A0). On May 5, 2020, the Reproductive Health Equity and Birth Justice Core of PRIORITY was launched to focus on recruitment of Black, Indigenous, and People of Color, expanding the enrollment mechanism to include referrals from partnering community-based organizations and the PRIORITY Community Advisory Board.

Enrollment was achieved through two mechanisms. Any participant could self-enroll through the PRIORITY website (http://priority.ucsf.edu). Alternatively, prenatal care clinicians (physicians or midwives) across the United States identified potential participants and provided them with information about the registry, Regardless of how the participant was referred, contact information of potential participants was provided to allow study staff at the coordinating center to contact them. After referral, participants underwent screening and eligible participants were contacted by a study coordinator by phone or email. Verbal or email consent was obtained per the institutional review board–approved protocol.

Participants will be followed for longitudinal data collection during the entire pregnancy and through 1 year after the pregnancy ends. Participants will complete self-reported questionnaires about demographic characteristics; medical and reproductive history; symptoms; and all pregnancy, obstetric, and neonatal outcomes (Appendix 1, available online at http://links.lww.com/AOG/C103). Participants may complete study questionnaires online through a secure portal or by phone, email, or on paper forms mailed to them. Literacy is not necessary to enroll, because participants can complete consent and all questionnaires verbally by phone with a trained research coordinator. Questionnaires are administered at baseline when participants enroll and every week for 4 weeks. If applicable, questionnaires will then be administered at 24 and 32 weeks of pregnancy and 6 weeks, 6 months, and 12 months postpregnancy. All data are collected and stored in a HIPAA-compliant database through REDCap (Research Electronic Data Capture).

At study enrollment, participants reported medical history and demographic information, including self-reported race and ethnicity based on categories used by the U.S. Census Bureau. Data on race and ethnicity were collected because of known disparities in maternal morbidity and mortality by race–ethnicity. Participants reported the date of SARS-CoV-2 testing, onset of symptoms, and whether they were in the hospital. Participants were asked to indicate the first symptom that they experienced from a list of symptoms and queried about which symptoms led them to undergo testing. During weekly follow-up for 4 weeks, participants were asked whether they had received SARS-CoV-2 test results and to indicate whether they were currently experiencing any known COVID-19 symptoms from a predefined list of symptoms. Participants could also mark “other” for symptoms not listed on the form and write in additional symptoms. For the current analyses, we used the date of symptom onset and the date of study enrollment combined with weekly self-reported symptoms to calculate the presence of symptoms for up to 8 weeks after symptom onset.

We adjudicated the accuracy of self-report of SARS-CoV-2 diagnosis through review of medical records and viral test results on a subsample of participants. Among polymerase chain reaction (PCR) test results for 140 participants, 138 (98.6%) were concordant with self-reports—119 PCR test results were positive for SARS-CoV-2 infection, and 19 were negative. Two participants (1.4%) reported that they tested negative for SARS-Co-V infection, but the PCR test result was positive several weeks before enrollment.

Participants were classified by self-reported SARS-CoV-2 infection status. If participants were enrolled in the study as a person under investigation, they were queried weekly about test results to place them into SARS-CoV-2–positive or SARS-CoV-2–negative groups. For this primary analysis, we included only those patients who tested positive for SARS-CoV-2 infection and reported COVID-19 symptoms at the time of testing. Participants with SARS-CoV-2 infection status that remained unknown 2 weeks after enrollment were excluded from the analysis (n=87).

We calculated summary statistics and associated 95% CIs. We used Kaplan-Meier survival analyses to estimate the time to resolution of symptoms in the subgroup of participants who reported any symptoms at the time of SARS-CoV-2 testing and had at least one follow-up visit. Statistical significance was set at *P*=.05. SAS 9.4, R 3.6.2, and Stata 15 were used for analysis.

## RESULTS

Participants included in this study were enrolled from March 22, 2020, until July 10, 2020 (Fig. 1). Of 736 participants, 594 tested positive for SARS-CoV-2 infection and 142 tested negative; only the former were included in the primary analysis. The cohort is demographically and clinically diverse (Table 1); 9% were enrolled with use of a medical interpreter. Most participants (67%) had contact with a person who was symptomatic or tested positive for SARS-CoV-2 infection, and 21% had a history of recent travel. Health care workers represent 31% of the cohort (Table 1). Baseline differences between the participants who tested positive for SARS-CoV-2 infection and those who tested negative are presented in Appendix 2, available online at http://links.lww.com/AOG/C104.

**Table 1.**
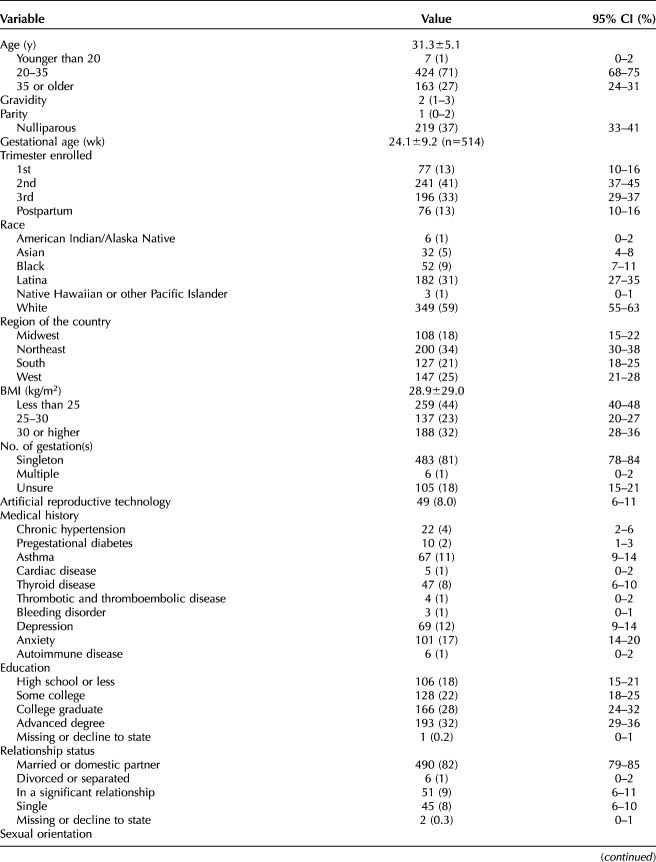

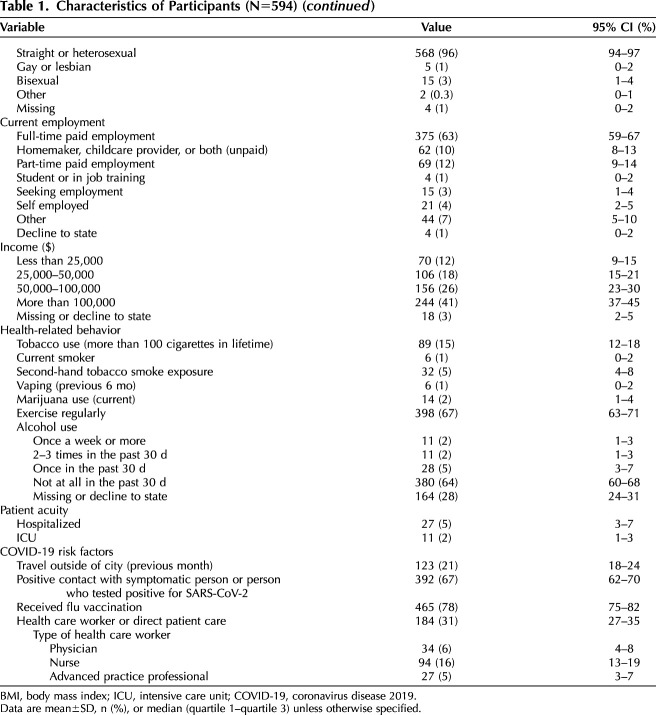
Characteristics of Participants (N=594)

The most common first symptoms reported by participants were cough (20%), sore throat (16%), body aches (12%), fever (12%), and headache (8%) (Table 2). The symptoms that led to testing were congruent with the top 10 first-presenting symptoms—among them, cough (61%), fatigue (51%), body aches (51%), headaches (46%), fever (44%), shortness of breath (40%), sore throat (39%), runny nose (34%), and anosmia or ageusia or both (32%). Differences in the clinical presentation and testing symptoms between participants who tested positive for SARS-CoV-2 infection and those who tested negative are presented in Appendix 3, available online at http://links.lww.com/AOG/C104.

**Table 2.**
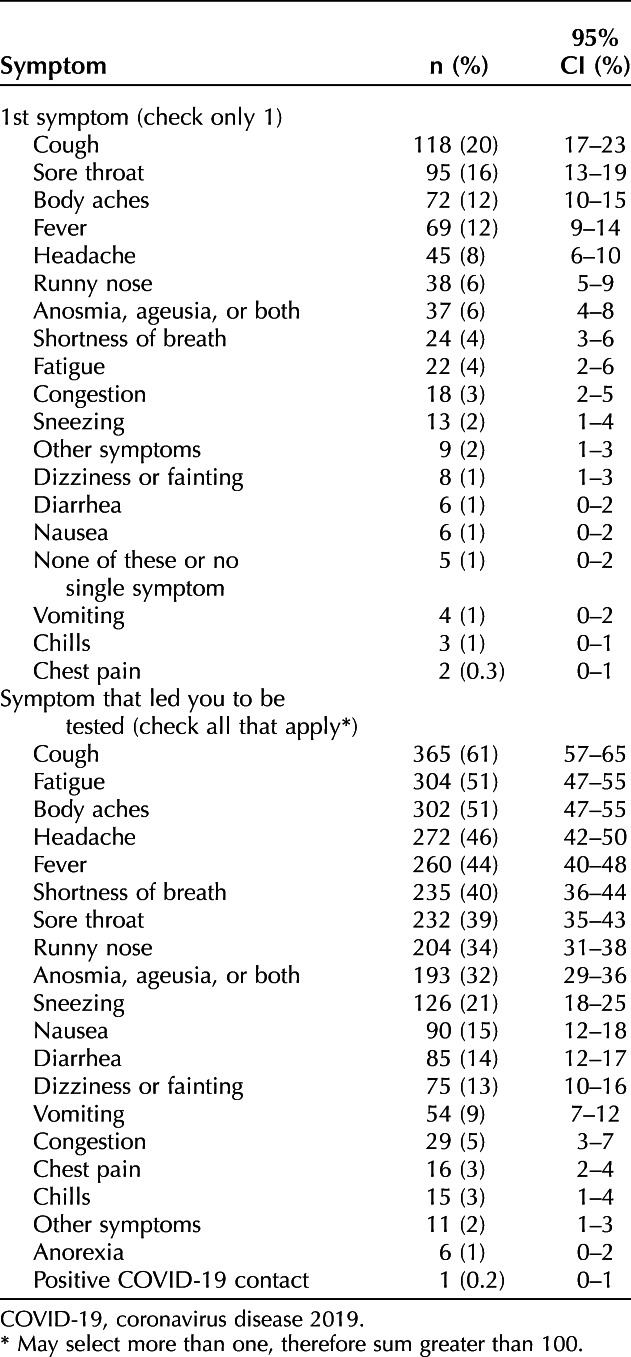
First Symptoms and Symptoms Leading to Testing (N=594)

**Fig. 2.**
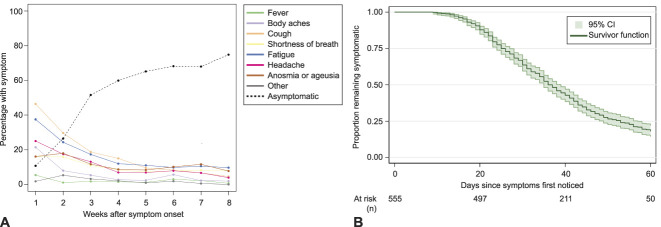
Symptoms over time (**A**) and resolution of symptoms (**B**) in patients who tested positive for severe acute respiratory syndrome coronavirus 2 (SARS-CoV-2) infection. Afshar. Clinic Presentation of COVID-19 in Pregnancy. Obstet Gynecol 2020.

The prevalence of all symptoms over time from week 1 after symptom onset to 8 or more weeks after the start of symptoms are listed in Table 3. Changes in the prevalence of the most common symptoms are shown in Figure 2A. The highest prevalence of symptoms was within the first 3 weeks after symptom onset.

**Table 3.**
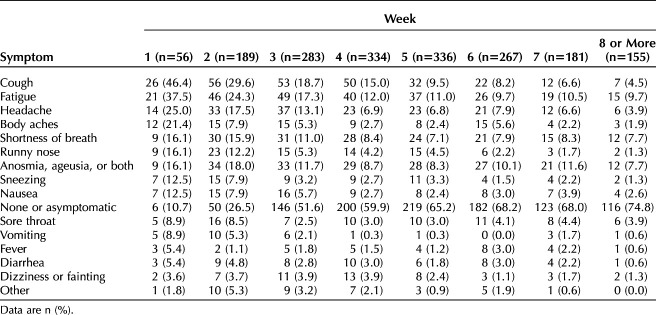
Clinical Course of Symptoms

Cough (46%), fatigue (38%), and headache (25%) were the most prevalent symptoms in the first week after diagnosis. Symptoms usually resolved within 1 month; by week 3, 52% of participants had become asymptomatic, and, by week 4, 60% were asymptomatic. By week 8, symptoms persisted in 25% of participants who tested positive for SARS-CoV-2 infection. The median time from any symptom onset to complete resolution of symptoms was 37 days (95% CI 35–39) (Fig. 2B). The change in symptoms over time among participants who tested negative for SARS-CoV-2 infection is depicted in Appendix 4, available online at http://links.lww.com/AOG/C104.

## DISCUSSION

As of July 2020, Centers for Disease Control and Prevention criteria for SARS-CoV-2 testing recommended symptom-based screening.^3^ We demonstrate that symptomatology related to COVID-19 is heterogenous and that this is further complicated by overlapping symptoms of normal pregnancy, including nausea, fatigue, and congestion, among others.

The prevalence of symptoms among participants who tested positive for SARS-CoV-2 infection was different than that in nonpregnant populations in prior publications. For instance, in a nonpregnant population–based cohort of 1,099 patients in China,^4^ the most common presenting symptoms were fever (43.8% on hospital admission) and cough (67.8%), compared with cough (20%) and sore throat (16%) as the most common first symptoms in our cohort, with fever present in only 12% of the population. One week after symptom onset among PRIORITY participants who tested positive for SARS-CoV-2 infection, cough (41%) and fatigue (33%) were prevalent but fever remained uncommon (5%). In a recent meta-analysis of 77 studies, pregnant patients with COVID-19 had a higher prevalence of fever (40%) as the presenting symptom than the prevalence of fever (12%) reported by PRIORITY participants. This difference in the presence of fever is likely because the meta-analysis primarily includes studies with hospitalized patients, who are more likely to be febrile, whereas 95% of PRIORITY participants are outpatients.

Of the group of participants who tested positive for SARS-CoV-2 infection, 31% are Latina and 9% are Black. As a volunteer registry of patients with known or suspected SARS-CoV-2 infection at the time of enrollment, our study design cannot estimate population incidence of infection. However, prior studies have found a higher incidence of and mortality from COVID-19 among Latino and Black populations across the country compared with White populations.^5,6^ Multiple factors may contribute to these racial and ethnic disparities in COVID-19 incidence and outcomes that have not been explored. The factors that lead to this discrepancy can to some degree be accounted for by historical and contemporary discrimination leading to economic disadvantages against people of color in the United States.^7^ To date, there have been demonstrated differences in disease prevalence and mortality from COVID-19 affecting Black and Latino communities.^8,9^ For instance, Latina individuals may have higher rates of employment in “essential” positions, preventing them from sheltering in place.

Despite pregnant patients having higher risk for severe disease with other respiratory viruses (H1N1, severe acute respiratory syndrome, Middle East respiratory syndrome), the data are less clear with SARS-CoV-2.^10,11^ Case series from China, the United States, and Sweden demonstrate the complete spectrum of lower, similar, and higher rates of morbidity in pregnant patients compared with the general population.^4,12,13^ A recent meta-analysis found an increased risk of admission to the intensive care unit and ventilation among pregnant patients with COVID-19 compared with nonpregnant patients. The majority of our study population had mild disease, as indicted by their nonhospitalized status. This is consistent with other studies, which have also demonstrated that the majority of pregnant patients with COVID-19 have mild disease.^14^ Known risk factors associated with severity of disease and poor outcomes with COVID-19 include medical comorbidities^15^; however, in our cohort, patients with diabetes, hypertension, and other comorbidities were not more likely to test positive for SARS-CoV-2 infection. We may have been underpowered to investigate these differences.

Strengths of this study include the prospective design with unique self-reported longitudinal follow-up of weekly symptoms during the first 4 weeks of enrollment. Lack of known timing for clinical presentation has been highlighted as an evidence gap in a recent meta-analysis.^16^ The large nationwide sample size recruited from every region of the country in a variety of practice settings is also a strength that supports generalization of study results. To our knowledge, this is the largest longitudinal cohort of ambulatory patients in the United States. However, this study does have limitations. Thirty percent of the study population are health care workers with associated high education level and income, and 60% of the population is White. This may limit generalizability of findings to the general population and to Latina, Black, and Native American populations, which have the highest incidence of and mortality from COVID-19.^17,18^ We are actively working with the PRIORITY Community Advisory Council and community-based organizations to increase enrollment of Latina, Black, and Native American patients. Other limitations include changes in testing availability over time based on participants' location and access during the study period, timing of testing relative to symptoms, and accuracy of testing, as discussed above, including both false-negative and false-positive results.^19,20^

It has been suspected that pregnant patients have a different clinical presentation of and morbidity from COVID-19 compared with the nonpregnant population. We demonstrate that the presenting symptoms in this primarily outpatient cohort of pregnant patients differ from those in nonpregnant populations, with a lower prevalence of fever and higher rates of fatigue, body aches, and headaches. Pregnancy also confers a prolonged course of disease for patients with COVID-19, where 25% of patients have persistent symptoms 8 weeks or more after disease onset.

**Authors' Data Sharing Statement**

Will individual participant data be available (including data dictionaries)? *No.*
What data in particular will be shared? *Not available.*
What other documents will be available? *Not available.*
When will data be available (start and end dates)? *Not applicable.*
By what access criteria will data be shared (including with whom, for what types of analyses, and by what mechanism)? *Not applicable.*

## Figures and Tables

**Figure:**
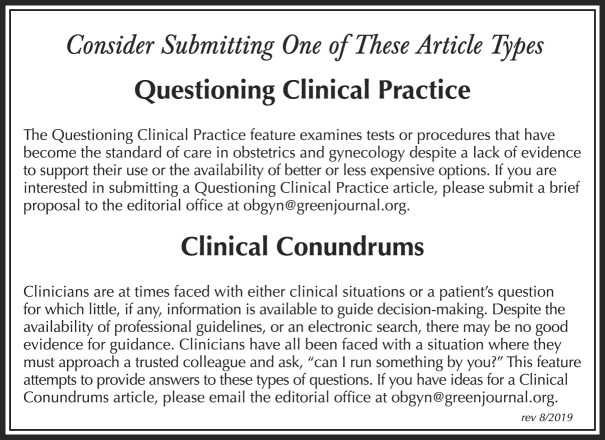
No available caption
